# Targeting the neutral hip-to-calcaneus axis in kinematically aligned total knee arthroplasty is feasible with fewer alignment outliers for varus osteoarthritic patients

**DOI:** 10.1007/s00167-023-07306-1

**Published:** 2023-03-15

**Authors:** Tomoyuki Matsumoto, Naoki Nakano, Kazunari Ishida, Toshihisa Maeda, Shotaro Tachibana, Yuichi Kuroda, Shinya Hayashi, Takehiko Matsushita, Ryosuke Kuroda

**Affiliations:** 1 Department of Orthopedic Surgery, Kobe University Graduate School of Medicine, 7-5-1, Kusunoki-cho, Chuo-ku, 650-0017 Kobe, Japan; 2 Department of Orthopedic Surgery, Kobe Kaisei Hospital, Kobe, Japan

**Keywords:** Total knee arthroplasty, Kinematic alignment, Mechanical alignment, Mechanical axis, Calcaneus, Ground mechanical axis

## Abstract

**Purpose:**

Assessment of the conventional mechanical axis (MA) (hip-to-talus axis) is reported to result in constitutional varus in the native knee. However, the ground MA (hip-to-calcaneus axis), which is the line from the hip center to the bottom of the calcaneus, passes through the center of the knee joint in the native knee and is a possible alternative target for total knee arthroplasty (TKA) assessments. Therefore, this study aimed to present a “ground kinematically aligned (KA)-TKA.” In this technique, the femoral component is placed on the cylindrical axis using the calipered technique and the tibial component is placed to give a neutral ground MA. Radiographical investigation was used to determine whether physiological alignment can be individually achieved with ground KA-TKA; this was compared with that of a tibia-restricted modified KA-TKA, referring to conventional MA (hip-to-talus axis) results.

**Methods:**

As the primary endpoint, this prospective cohort study compared the ground MA ratios of the knee joints in 40 ground KA-TKAs (G group: Coronal Plain Alignment of the Knee (CPAK) 28 type I, 7 II, 1 IV, and 4 V) with those of the preceding 60 modified KA-TKAs (M group: CPAK 46 type I, 12 II, and 2 V) performed for patients with varus osteoarthritis (OA). The number of outliers differing over ± 5% from the neutral were compared between groups using the χ^2^-test. The Hip–knee–ankle (HKA) angle, coronal femoral/tibial component alignment (FCA/TCA), and joint line orientation angle (JLOA) were compared between the groups using non-paired *t-*tests. Statistical significance was set at *p* < 0.05.

**Results:**

The G group had a higher ratio of the ground MA passing through the knee center than the M group did; outliers differing over ± 5% from the neutral of the ground MA were 2/40 cases in the G group and 20/60 cases in the M group, which was a significant difference (*p* = 0.001). The HKA angle, FCA/TCA, and JLOA were not significantly different between the groups.

**Conclusions:**

Targeting the ground MA in KA-TKA for patients with varus OA was feasible and has the potential to provide a physiological alignment more similar to the native knee in TKA than other kinematic alignment techniques.

**Level of evidence:**

Level III.

## Introduction

Recently, the alignment philosophy underpinning the study of total knee arthroplasty (TKA) has tended to shift from mechanically aligned TKA as the gold standard to personalized alignment instead [10]. Hirschmann et al. created a new classification for functional knee phenotypes using a coronal lower limb alignment based on the native alignment in young individuals without osteoarthritis (OA) [12]. Based on 125 possible functional knee phenotypes, they indicated the eight most common functional phenotypes which covered two-thirds of the total population and represented which phenotypes were suitable for mechanical, anatomical, and restricted kinematic alignment. The group also confirmed the great variability of joint line orientation in osteoarthritic and non-osteoarthritic knees by assessing the femoral mechanical angle (FMA) and tibial mechanical angle (TMA), indicating the necessity of a more individualized approach in TKA [9, 11]. More recently, MacDessi et al. introduced the Coronal Plane Alignment of the Knee (CPAK) classification system which classified knee phenotypes based on constitutional limb alignment and joint line obliquity [21]. The classification system also indicated that the kinematic approach was suitable for Type I (varus, apex distal joint line) and type IV (varus, neutral joint line) out of nine classification categories. Anatomical and restricted kinematically aligned (KA)-TKA [14, 16] have gained popularity for reproducing physiological joint lines and kinematics with minimal soft tissue release, and achieve better clinical outcomes than mechanically aligned TKA. However, recent meta-analyses have shown that the advantage of KA-TKA is still controversial compared with mechanically aligned TKA; one showed better early clinical outcomes and another did not [4, 7, 13].

The concept of constitutional varus, which indicates that a young native knee is not always in the neutral mechanical axis (MA) but slightly varus [1], is one of rationales for KA-TKA procedures. This concept raises the question of why these young healthy knees are not in the neutral MA. Haraguchi et al. suggested that true MA, previously known as the ground MA [5, 26], should be assessed from the hip center to the lowest point of the calcaneus, rather than the ankle center [8]. Recently, full-length leg assessment, including that of the calcaneus, has attracted attention as an alternative alignment assessment [17, 19, 20, 27]. Following this concept, Matsumoto et al. reported on a tibia-restricted modified KA-TKA procedure [23], in which the femoral component was placed on the cylindrical axis using the calipered technique, and the tibial component was constantly placed at 3° varus. On average, this resulted in joint lines parallel to the ground and a similar alignment to that in young healthy individuals [31], with the ground MA (hip-to-calcaneus axis) unexpectedly passing through the center of the knee joint. Furthermore, Kamenaga et al. performed a gait analysis and reported that plantar pressure distribution after the tibia-restricted modified KA-TKA, not mechanically aligned TKA, is similar to that in normal individuals [18]. Hence, for individualized and constant reproduction of native limb alignment and knee kinematics [24], the ground MA (hip-to-calcaneus axis) may be an alternative target, especially for KA-TKA.

Tibia-restricted modified KA-TKA, in which the tibial bone cut is performed with a systemic 3° varus, does not completely follow the kinematic alignment technique [14, 15]. However, this KA-TKA does occasionally, but not intentionally, achieve neutral ground MA on average, despite not targeting the ground MA (hip-to-calcaneus axis) [23]. Therefore, this study aimed to present the ground KA-TKA for patients with varus OA. In this technique, the femoral component is placed on the cylindrical axis using the calipered technique and the tibial component is placed to give a neutral ground MA (hip-to calcaneus axis), then the postoperative radiographic parameters in ground KA-TKA are compared with those in tibia-restricted modified KA-TKA. The hypothesis of the study was that this ground KA-TKA technique would reproduce a neutral ground MA (hip-to-calcaneus axis) with fewer alignment outliers compared with tibia-restricted KA-TKA, because the ground KA-TKA technique individually fits the anatomical differences of each knee.

## Materials and methods

### Radiographic simulation for the ground KA-TKA technique

**Fig. 1:**
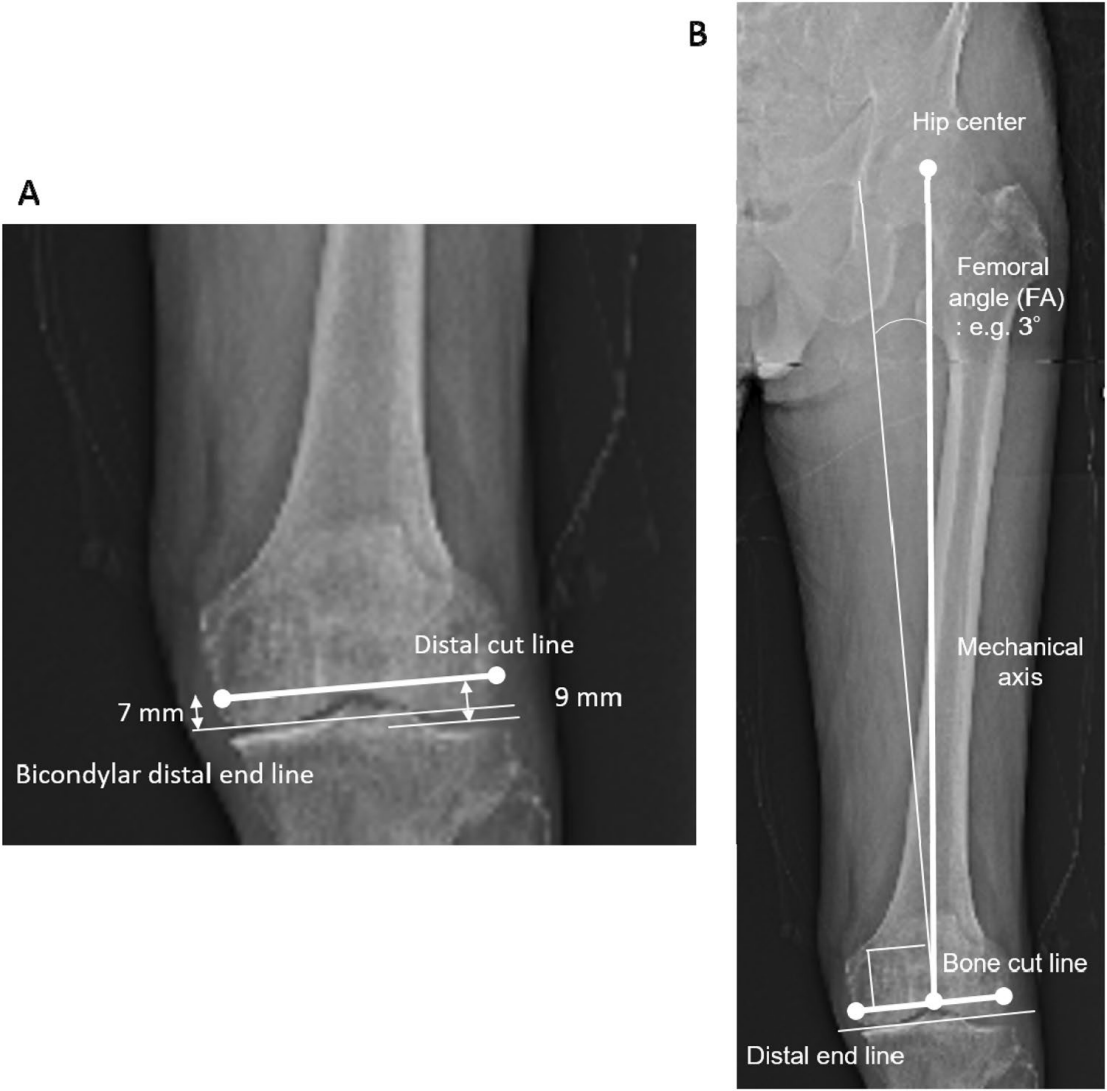
Distal femoral bone cut simulation. **A** The femoral bone cut line was simulated to be 7 mm proximal to and parallel to the bicondylar distal end line. Considering a 2-mm cartilage thickness, the medial side with cartilage wear and the lateral side with invisible cartilage were 7 mm each (equal to a 9-mm distal thickness of the femoral component). **B** The mechanical axis from the hip center to the center of the distal cut line generally results in valgus in relation to the perpendicular line of the distal cut line. In this case, the femoral angle is 3° valgus

To simulate ground KA-TKA, the femoral distal cut line and tibial proximal cut line were first simulated using full-length standing coronal radiography that included the calcaneus. The femoral distal cut was 9 mm thick at the lateral side and 7 mm thick at the medial side, as per the calipered technique. Considering the cartilage thickness, the distal cut line was simulated to be 7 mm proximal to both the medial and lateral sides of the bicondylar distal end line (Fig. 1A). The MA of the femur was from the hip center to the center of the distal cut line. The femoral angle (FA), which is commonly valgus in varus-type OA, was defined and measured as the angle between the MA and the line perpendicular to the distal cut line(Fig. 1B).

**Fig. 2:**
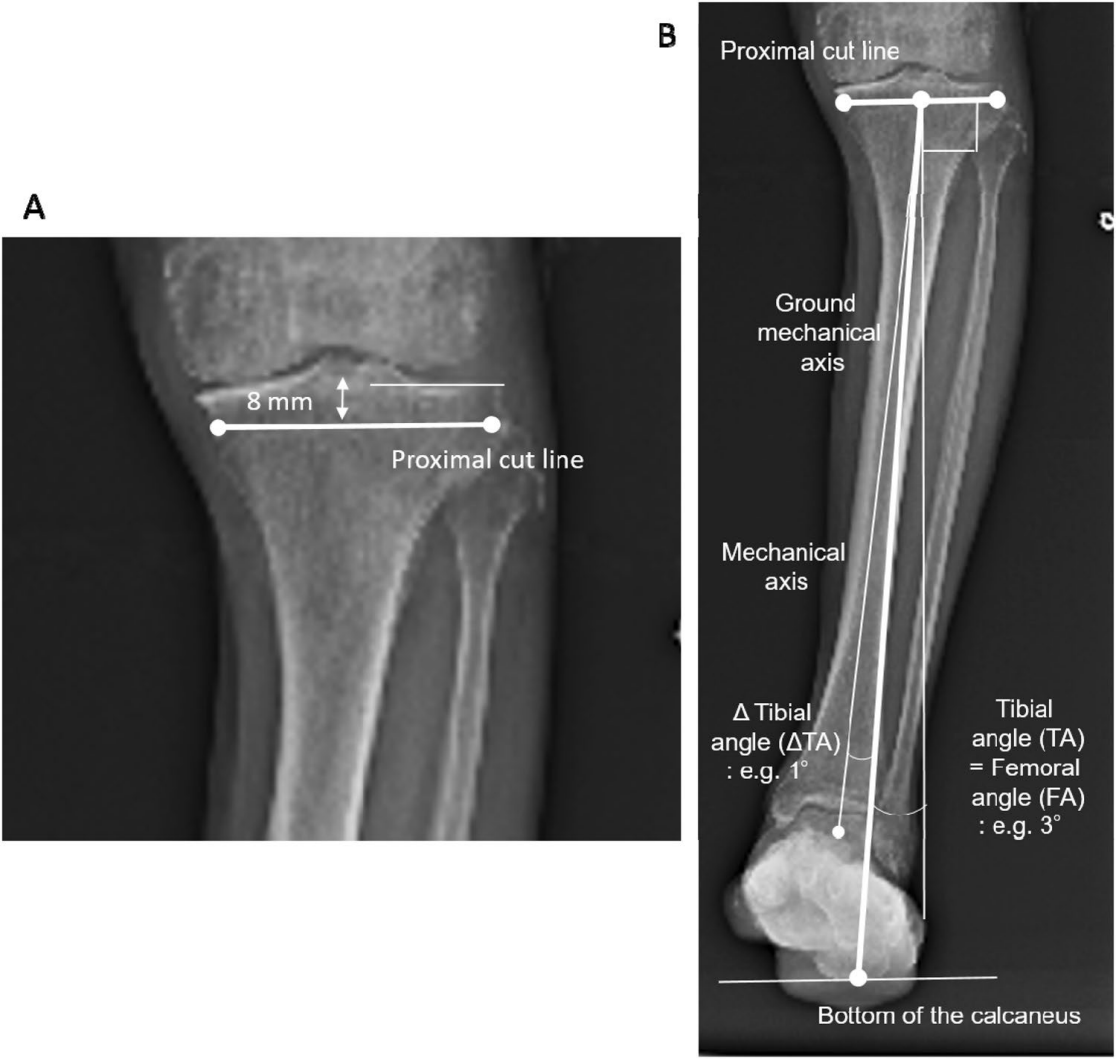
Proximal tibial bone cut simulation. **A** The proximal cut line was 8 mm distal to the lateral joint line of the tibia. Considering a 2-mm cartilage thickness, the lateral side with invisible cartilage was 8 mm (equal to a 10-mm thickness of the tibial component). **B** The proximal cut line was simulated to neutralize the femoral angle in relation to the ground mechanical axis from the center of the proximal cut line to the bottom of the calcaneus. The navigation system refers to the mechanical axis and not ground mechanical axis. The mechanical axis is typically medial to the ground mechanical axis. If the ankle is located 1° medial to the calcaneus (ΔTA = 1° varus) and the FA is 3° valgus (TA = 3° varus), the navigated tibial cut angle should be 4° varus

For simulation of the tibial side, considering the 2-mm thickness of the cartilage, the proximal cut line was 8 mm distal to the lateral joint line of the tibia (Fig. 2A). The proximal cut line was set to neutralize the FA in relation to the ground MA from the center of the proximal cut line to the bottom of the calcaneus. If the resulting FA was 3° valgus to the MA of the femur, a 3° varus tibial cut in relation to the ground MA of the tibia as the tibial angle (TA) was performed with the assistance of the navigation system. The navigation system referred to the ankle center rather than the bottom of the calcaneus. Therefore, the ΔTA was defined as the angle between the MA and ground MA of the tibia and was measured preoperatively. Generally, the calcaneus is located lateral to the ankle center. If the FA were 3° valgus (TA = 3° varus) and the bottom of the calcaneus 1° lateral to the ankle center (ΔTA = 1° varus), the navigated tibial cut angle (nTA) would be 4° varus (Fig. 2B). The calculation of these parameters was as follows: nTA (varus) = TA (varus)(FA (valgus)) + ΔTA (varus).

### Ground KA-TKA compared with modified KA-TKA

**Fig. 3:**
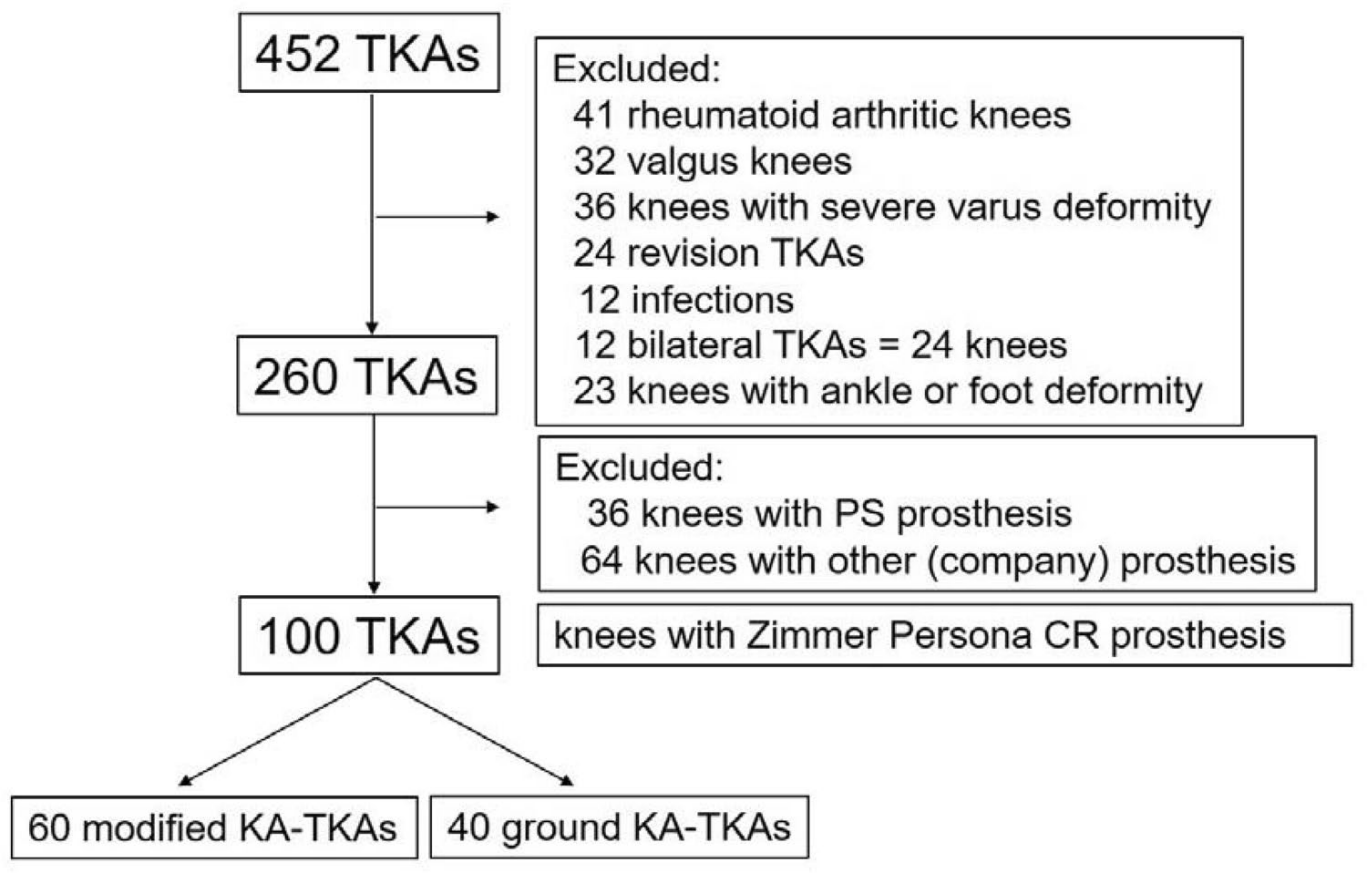
Flowchart of patients undergoing each type of total knee arthroplasty. KA-TKA: kinematically aligned total knee arthroplasty; PS: posterior-stabilized; CR: cruciate-retaining

The hospital’s ethics committee approved the study protocol (Kobe University, No. 290038), and the patients provided written informed consent for participation in the study. The inclusion criteria were substantial pain and loss of function due to severe OA of the knee (Kellgren–Lawrence grade 3–4), with a functional posterior cruciate ligament (PCL) based on the preoperative epicondylar view radiograph and CT of intercondylar osteophytes. To make a fair assessment and minimize the influences of clinical variables, the exclusion criteria were knees with valgus deformity, severe varus deformity > 20°, flexion contracture > 20°, revision TKAs, active knee joint infections, or the need for bilateral TKA. To avoid compensatory hindfoot alignment change postoperatively [3, 28], patients with prior ankle or foot surgery, foot or ankle deformity (such as flat foot, hallux valgus, and ankle OA), history of ankle fracture, and those unable to stand stably on one leg for > 10 s without support were also excluded. From January 2019 to December 2021, 100 consecutive patients meeting the abovementioned criteria were prospectively enrolled in this study and underwent cruciate-retaining TKA (Persona®. Zimmer Inc., Warsaw, IN, USA) using a portable navigation system (iASSIST® Zimmer-Biomet Japan Inc., Tokyo, Japan) (Fig. 3). This cohort included 60 consecutive tibia-restricted modified KA-TKAs performed from January 2019 to December 2020 (group M), and 40 consecutive ground KA-TKAs (group G) performed from January to December 2021. All operative procedures were performed by a senior surgeon (T.M.) with > 15 years of experience in performing TKAs. The patients’ demographic data, including age, sex, body mass index, and preoperative deformities, demonstrated no significant differences between the groups (Table 1).

**Table 1:** Patient demographic data

	Ground KA-TKA	Modified KA-TKA	*p*-value
Age (years)*	75.8 (56–86)	75.0 (57–91)	0.971
Sex (% male)	15.0% (6/40)	16.7% (10/60)	> 0.999
Body mass index**	27.3 ± 4.9	26.2 ± 3.2	0.384
Deformity (varus) (degree)**^#^	10.8 ± 2.5	12.0 ± 4.8	0.147

KA-TKA: kinematically aligned total knee arthroplasty

*Data are presented as mean (range)

**Data are presented as mean ± standard deviation

^#^Positive values indicate varus alignment

#### Operative procedures

After inflating the air tourniquet to 250 mmHg, medial parapatellar arthrotomy was performed. All surgeries were performed using the extension-gap-first technique. Following the confirmation of functional PCL based on intraoperative findings, the PCL insertion was preserved by creating a bony island.

For the ground KA-TKAs, the distal femoral cut was performed with the assistance of a portable navigation system, followed by the tibial cut using the calipered technique [15]. Before femoral osteotomies, minimum medial release (osteophyte removal and release of the deep layer of the medial collateral ligament) was performed to maintain medial stability [25]. Femoral osteotomies were performed after correcting for cartilage wear from the distal and posterior femoral condyle equal in thickness (9 mm) to the femoral component; the rotational angle of the femur relative to the posterior condylar axis was set as 0° [23]. Based on the FA value, which was confirmed by preoperative planning and the navigation system, the nTA value was determined by targeting the neutral ground MA, as planned preoperatively. Thus, distal femoral and proximal tibial cuts were performed by referring to the preoperative simulation and the navigation value.

For the tibia-restricted modified KA-TKAs, the femoral cut was made using the same method as the ground KA-TKA, with the assistance of the portable navigation system. Tibial osteotomy was performed 3° varus in relation to the MA and the original posterior slope (up to 10°). Based on a previous report wherein the tibial plateau inclination was approximately 3° in asymptomatic volunteers regardless of age, but progressed to approximately 10° with OA progression [22], 3° varus was applied to avoid severe varus tibial implantation.

#### Radiographic measurement

Preoperatively and 1 month postoperatively, full-length standing coronal radiographs that included the calcaneus (hip-to-calcaneus radiograph) were obtained to evaluate the ground MA, as previously described [23, 29]. The patient maintained a unipedal stance on a radiolucent platform and faced a long film cassette. For the lowest point of the calcaneus to be visualized by radiography, the cassettes must slide into a position where the lower edge passes through the edge of the platform. The patient’s patella should be placed forward and ankle position should be neutral. The X-ray beam was centered on the knee of the imaged leg from a distance of 2 m. The voltage and current were 200 mA and 85 kV, respectively. It was important to confirm on the radiograph that the patella was centered between the femoral condyles and that the ankle was placed in the neutral position. When lift-off of the femoral component from the tibial insert was found on either the medial or lateral side, the radiograph was re-taken to achieve equal weight-bearing on both medial and lateral sides.

Preoperatively, the lateral distal femoral angle (LDFA), defined as the angle formed by the femoral MA and the joint line of the distal femur on the lateral side, and the medial proximal tibial angle (MPTA), defined as the angle formed by the tibial MA and the joint line of the proximal tibia on the medial side, were measured. The arithmetic hip–knee–ankle angle (aHKAA) and joint line obliquity angles (aJLOA) were measured in accordance with MacDessi et al. (aHKAA = LDFA – MPTA, aJLOA = LDFA + MPTA) [21]. Knee phenotypes were based upon the coronal plane alignment of the knee (CPAK) classification, which grouped patients into nine categories based on their aHKAA (varus, neutral, valgus) and aJLOA (apex distal, apex neutral, and apex proximal).

**Fig. 4:**
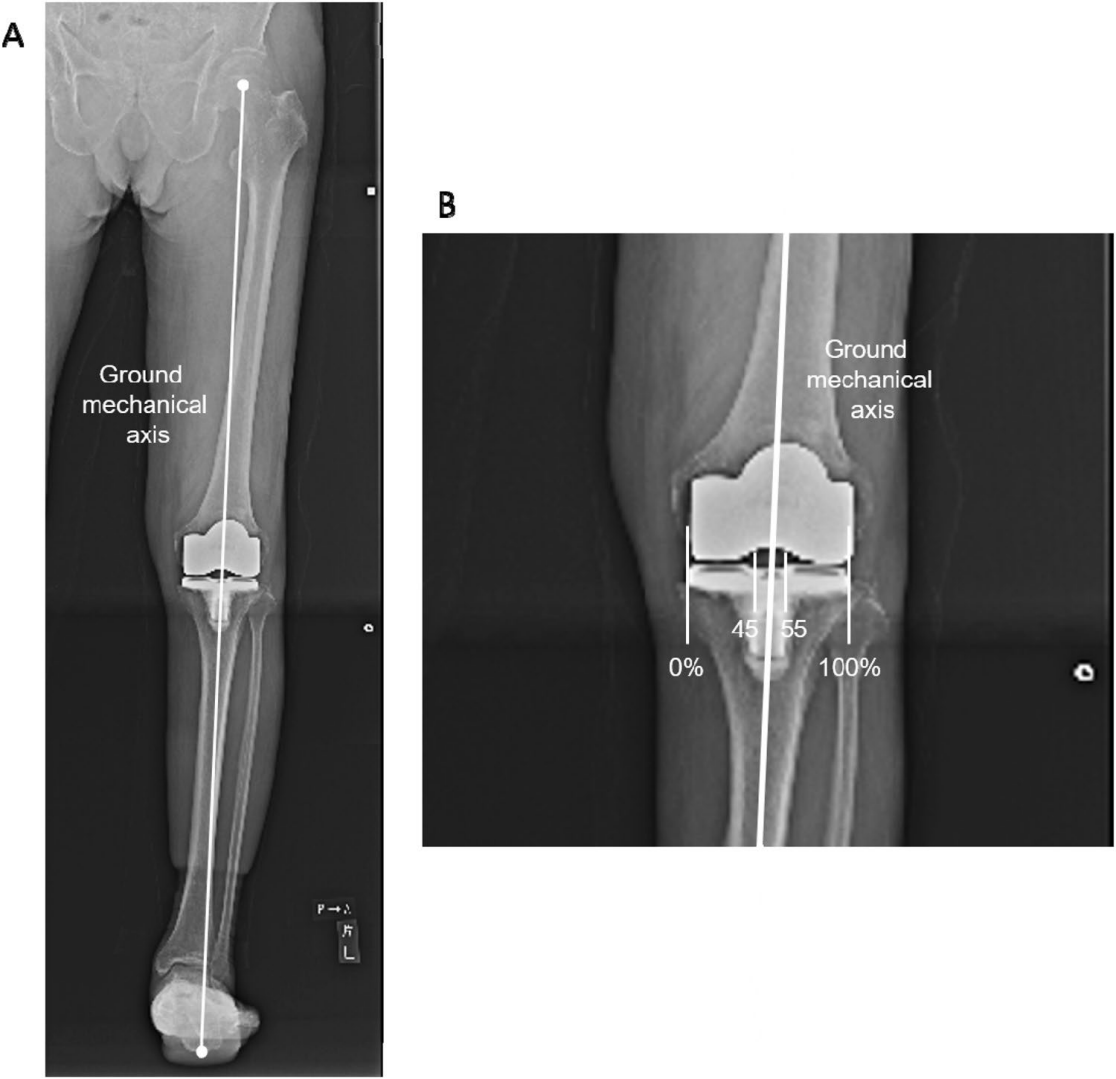
Assessment of ground mechanical axis. **A** The ground mechanical axis from the hip center to the lowest point of the calcaneus was assessed. **B** The ground mechanical axis ratio of the knee joint and outliers from the neutral within ± 5% were assessed

Postoperatively, the HKA angle, coronal femoral/tibial component alignment (FCA/TCA), and joint line orientation angle (JLOA) to the ground during one-leg standing were compared between the groups. The ground MA (the line from the hip center to the lowest point of the calcaneus) ratios of the knee joint were compared between the groups (Fig. 4A). To measure the ground MA, the medial and lateral edges of the tibial plafond were considered as 0% and 100%, respectively. The number of outliers from the neutral within ± 5% were also compared between the groups (Fig. 4B).

To determine the intra- and inter-observer reliabilities of the radiographic assessments, the two investigators performed all radiographic assessments twice on 20 randomly selected radiographs. The intra- and inter-observer reliabilities of all radiographic measurements were evaluated using intraclass correlation coefficients (ICCs). The ICCs for intra- and inter-observer reliability were > 0.85 (range, 0.85–0.96) for all measurements. Based on the observed reliability of the results, measurements obtained by only one of the investigators (S.T.) were used in the analyses.

#### Statistical analysis

All values are normally distributed and expressed as mean ± standard deviation (SD). The results were analyzed using a statistical software package (Graph Pad Prism software, Graph Pad, California, USA). The numbers of outliers between the groups were analyzed using the χ^2^-test. Non-paired *t-*tests were used to compare parameters between the groups. Statistical significance was set at *p* < 0.05. Power analysis was performed using G*Power 3 (Heinrich Heine, University of Dusseldorf, Dusseldorf, Germany) [6]. Based on a preliminary study comparing ground and modified KA-TKA using the χ^2^-test (outliers: 2/20 in ground KA-TKA and 4/20 in modified KA-TKA), the effect size was calculated as 0.333. Using a prior power analysis, an estimated sample size of 71 patients was required with a power (1-β) of 0.80, a type I error (α) of 0.05, and a calculated effect size. With a sample of 100 patients, the study would have a power (1-β) of 0.92 using the same method as described above.

## Results

### CPAK classification of knee phenotype

Preoperative knee phenotypes based on CPAK classification in this study were 28 type I, 7 type II, 1 type IV, and 4 type V cases out of 40 in the ground KA-TKA group, and 46 type I, 12 type II, and 2 type V cases out of 60 in the modified KA-TKA group.

### Radiographic simulation for ground KA-TKA technique

The simulated FA and ΔTA of 40 ground KA-TKAs were 1.8 ± 1.5° valgus and 0.7 ± 0.7° varus, respectively.

### Ground KA-TKA compared with modified KA-TKA

**Fig. 5:**
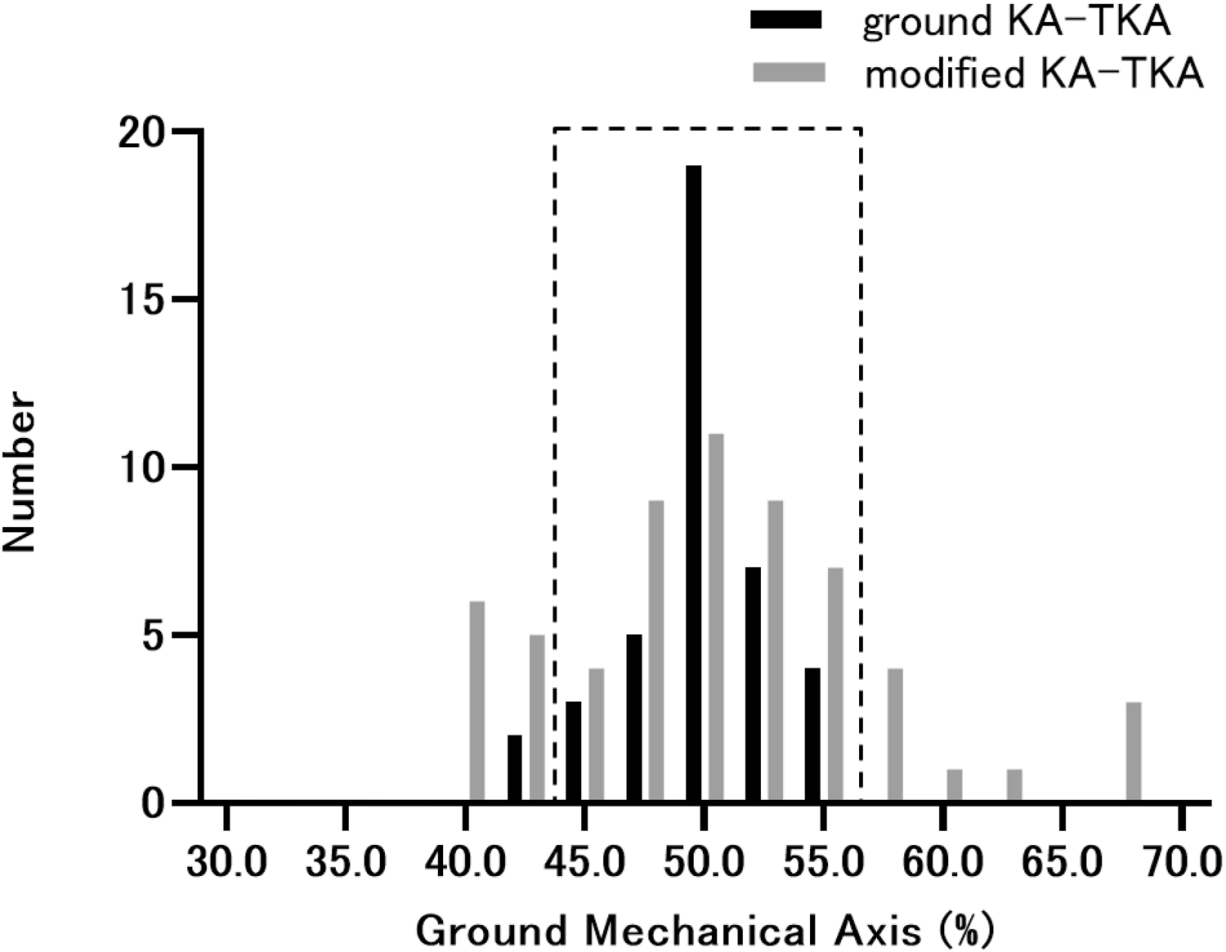
Histogram of ground mechanical axis. The ground mechanical axes passing through the neutral within ± 5% (surrounded by the dotted line) were 95.0% in the ground KA-TKA and 66.6% in the tibia-restricted modified KA-TKA. KA-TKA: kinematically aligned total knee arthroplasty

The mean ground MA ratios of the knee joints were 50.0 ± 2.0% in the G group and 50.5 ± 6.6% in the M group, with no significant difference. The numbers of outliers from the neutral ground MA within ± 5% were 2/40 cases (5.0% outliers) in the G group and 20/60 cases (33.3% outliers) in the M group, which had a significant difference (*p* = 0.001). A histogram of the ground MA ratio of the knee joint is presented in Fig. 5.

The postoperative radiographic parameters are listed in Table 2. The HKA angle, FCA/TCA, and JLOA were not significantly different between the groups.

**Table 2:** Postoperative radiological parameters

	Ground KA-TKA	Modified KA-TKA	*p*-value
HKA angle (°)	0.8 ± 1.0 varus (4.0 valgus–3.5 varus)	1.2 ± 1.6 varus (4.0 valgus–5.0 varus)	0.178
FCA (°)	1.7 ± 1.8 valgus (4.0 valgus–2.0 varus)	1.5 ± 1.3 valgus (4.0 valgus–2.0 varus)	0.587
TCA (°)	2.5 ± 1.5 varus (1.5 valgus–4.5 varus)	2.9 ± 1.2 varus (2.0 valgus–5.5 varus)	0.181
JLOA (°)	0.6 ± 1.8° varus (3.6 valgus–4.3 varus)	0.9 ± 2.0° varus (2.3 valgus–5.3 varus)	0.445

Data are presented as mean ± standard deviation (range). HKA: hip–knee–ankle; FCA: femoral component alignment; TCA1: tibial component alignment; JLOA: joint line orientation angle; KA-TKA: kinematically aligned total knee arthroplasty

## Discussion

The most important finding of this study was that for patients with varus OA, ground KA-TKA achieved fewer alignment outliers from the neutral ground MA than tibia-restricted modified KA-TKA did, as hypothesized. In KA-TKA, the physiological joint line and kinematics are ideal for reproducing the pre-arthritic knee. However, pre-arthritic knees and young healthy knees demonstrate slight varus alignment on average in conventional MA assessment [1, 31], but neutral alignment on average in the ground MA assessment [26, 31]. Thus, our philosophy of targeting the neutral ground MA in KA-TKA is reasonable. To the best of our knowledge, this study is the first to present the concept and feasibility of this new KA-TKA methodology that targets the neutral ground MA.

The ankle and hindfoot should not be ignored in the evaluation of knee OA, although conventional MA (hip-to-talus axis) does not take these into account. Thus, assessment of the ground MA (hip-to-calcaneus axis) in TKA is considered important. Tanaka et al. reported on 34 healthy individuals (mean age, 26.4 years) whose ground MA ratios were 51.4% and 50.4% and conventional MA ratios were 46.3% and 46.1% in single-leg and double-leg standing positions, respectively. This indicated that the ground MA should be taken into consideration for the assessment or planning of knee osteotomy and reconstruction surgeries [31]. Ishii et al. reported that the bottom of the calcaneus was lateral to the center of the ankle in 88.3% (113/128 knees) of knee OA patients; a 1.2° difference on average between the ground and conventional MA of the tibia [17]. Furthermore, Kikuchi et al. reported that in 21 varus OA patients the ground MA was more closely correlated with knee adduction angular impulse than the conventional MA [19]. Considering these previous reports, ground MA (hip-to-calcaneus axis) is a potential alternative target for knee surgeries.

When targeting the ground MA in TKA, foot and ankle joint conditions should be considered. Chandler and Moskal evaluated knee and hindfoot alignment before and after TKA and concluded that after changes in knee alignment, hindfoot alignment usually changed in relation to the degree of preoperative hindfoot deformity [2]. In a retrospective study, Norton et al. identified compensatory hindfoot alignment after TKA and where it occurred among those with end-stage OA undergoing TKA [28]. In a recent review article, Naylor et al. summarized the potential involvement of the ground KA as the evaluation axis when considering both hindfoot and knee alignment changes after TKA [27]. In the current study, ankle/foot deformities were excluded to avoid compensatory hindfoot alignment changes. In future studies, despite the lower influence on hindlimb alignment change in the present study, long-term radiological assessment after the ground KA-TKA should be investigated.

This study has several limitations. First, the assessments were performed for a small patient population, and those with severe varus, valgus deformities, and ankle/foot deformities were excluded. In this study population, approximately 75% were CPAK type I (varus, appendix distal joint line). Schelker et al. reported in a systemic review that the “safe zone” of ± 3° derived from the mechanical alignment strategy is hardly applicable for all knee phenotypes, so more modern alignment strategies and evidence-based thresholds for new patient-specific alignment strategies were urgently required [30]. In the future, suitable knee phenotypes including Hirschmann’s [12] and MacDessi’s [21] classifications for ground KA-TKA should be investigated by widening the patient population. The radiological two-dimensional simulation of the surgery was another limitation of this study. The influence of limb rotational position on parameter changes should be validated by three-dimensional analysis. Most importantly, the clinical outcomes were not assessed. Reduced alignment outliers in ground KA-TKA may lead to good clinical outcomes without any catastrophic failures; however, its clinical relevance should be investigated further in the future.

## Conclusions

In conclusion, the ground KA-TKA technique with radiological preoperative planning was easily feasible for mild-to-moderate varus OA patients. This new KA-TKA procedure, as a personalized alignment technique, may provide greater physiological alignment which is more comparable to the native knee than other alignment techniques in TKA.

## Data Availability

The data that support the findings of this study are available on request from the corresponding author.
